# Glyphosate Results Revisited

**DOI:** 10.1289/ehp.113-1257613

**Published:** 2005-06

**Authors:** Donna R. Farmer, Timothy L. Lash, John F. Acquavella

**Affiliations:** Product Safety Center, Monsanto Company, St. Louis, Missouri, E-mail: donna.r.farmer@monsanto.com; Boston University School of Public Health, Boston University, Boston, Massachusetts; Product Safety Center, Retired, Monsanto Company, St. Louis, Missouri

With respect to the recent article by De Roos et al. (2005), we would like to *a*) comment on the authors’ incomplete genotoxicity review, which is inconsistent with conclusions reached by regulatory agencies; *b*) estimate the likely range of systemic doses and margins of exposure for farmers based on comprehensive glyphosate biomonitoring data published in 2004; and *c*) request further evaluation of confounding and selection bias in their analyses for multiple myeloma.

In their discussion of genotoxicity, De Roos et al. focused on selected studies that conflict with the weight of evidence for glyphosate and Roundup brand (Monsanto Company, St. Louis, MO) agricultural herbicides containing glyphosate. They cited Williams et al. (2000) regarding the lack of a carcinogenic effect in rodent feeding studies with glyphosate but neglected to cite the extensive genotoxicity review in the same article in which Williams et al. concluded that Roundup and its components do not pose a risk for heritable or somatic mutations. This conclusion is in agreement with findings by the U.S. Environmental Protection Agency (U.S. EPA 1993), the World Health Organization (WHO 1994), the European Commission (2002), and regulatory agencies worldwide. None of the studies cited by De Roos et al. (2005) as presumptive evidence of genotoxicity were conducted under Good Laboratory Practices or according to international guidelines. Additionally, many of these studies used toxic dose levels and/or irrelevant routes of exposure.

When evaluating epidemiologic findings, it can be helpful to compare the range of likely exposure levels to the exposure levels of toxicologic significance (Acquavella et al. 2003). The cancer no-effect levels for glyphosate, based on rat and mouse lifetime feeding studies, are 1,000 and 1,500 mg/kg/day, respectively (Williams et al. 2000). Acquavella et al. (2004) reported results of a biomonitoring study in which 48 farmers collected all of their urine over 5 consecutive days (before, during, and for 3 days after a glyphosate application). In this study, the maximum systemic dose resulting from application of glyphosate to areas as large as 400 acres was 0.004 mg/kg. The geometric mean systemic dose was 0.0001 mg/kg. Accordingly, in the worst-case situation, if a farmer made a similar application every day for a lifetime, the systemic dose would be at least 250,000-fold lower than the cancer no-effect level in rodents. Indeed, this very large margin of exposure combined with the lack of evidence for genotoxicity must be factored into an assessment of biologic plausibility.

Finally, De Roos et al.’s Table 2 (De Roos et al. 2005) shows an age-adjusted relative risk (RR) of 1.1 [95% confidence interval (CI), 0.5–2.4] associating multiple myeloma and ever-use of glyphosate. The RR adjusted for selected demographic and lifestyle variables was 2.6 (95% CI, 0.7–9.4). The factors that account for the difference in these RRs are not well explained. Given the weak associations between the covariates and ever-use of glyphosate and the weak or nonexistent relation between these variables and risk of multiple myeloma, it is unlikely that the change in RR from 1.1 to 2.6 is attributable to confounding. The authors mention that only 75% of eligible subjects were included in the fully adjusted analysis and that this reduction in analytic sample size was due to the exclusion of subjects that were missing covariate data. Further, De Roos et al. (2005) did not find an association in the complete data set without adjustment for covariates (RR = 1.1), but they did find a positive association in the restricted data set without adjustment for covariates. The difference in association due simply to restricting the data set to those with covariate information was not quantified, although such quantification would help the reader understand what proportion of the change from 1.1 to 2.6 was attributable to adjustment for candidate confounders and what proportion was due to selection of subjects with more complete data. An analysis stratified by each covariate individually should have allowed the investigators to identify covariates for which missing data and/or adjustment made the biggest impact on the estimated RR. The identity of these covariates would help the reader weigh the potential for confounding versus selection bias to explain the change in RR from 1.1 to 2.6. Given that only 32 cases of multiple myeloma were observed and as few as 19 cases were included in some of the analyses, the authors should have explored the potential for the analysis of sparse data to result in estimates biased away from the null (e.g., see Greenland et al. 2000 for an example involving conditional logistic regression).
